# Differences in the Delivery of Health Education to Patients With Chronic Disease by Provider Type, 2005–2009

**DOI:** 10.5888/pcd11.130175

**Published:** 2014-03-06

**Authors:** Tamara S. Ritsema, Jeffrey B. Bingenheimer, Patty Scholting, James F. Cawley

**Affiliations:** Author Affiliations: Jeffrey B. Bingenheimer, James F. Cawley, The George Washington University, Washington, DC; Patty Scholting, The University of Nebraska Medical Center, Omaha, Nebraska

## Abstract

**Introduction:**

Health education provided to patients can reduce mortality and morbidity of chronic disease. Although some studies describe the provision of health education by physicians, few studies have examined how physicians, physician assistants, and nurse practitioners differ in the provision of health education. The objective of our study was to evaluate the rate of health education provision by physicians, physician assistants, and nurse practitioners/certified midwives.

**Methods:**

We analyzed 5 years of data (2005–2009) from the outpatient department subset of the National Hospital Ambulatory Medical Care Survey. We abstracted data on 136,432 adult patient visits for the following chronic conditions: asthma, chronic obstructive pulmonary disease (COPD), depression, diabetes, hyperlipidemia, hypertension, ischemic heart disease, and obesity.

**Results:**

Health education was not routinely provided to patients who had a chronic condition. The percentage of patients who received education on their chronic condition ranged from 13.0% (patients with COPD or asthma who were provided education on smoking cessation by nurse practitioners) to 42.2% (patients with diabetes or obesity who were provided education on exercise by physician assistants). For all conditions assessed, rates of health education were higher among physician assistants and nurse practitioners than among physicians.

**Conclusion:**

Physician assistants and nurse practitioners provided health education to patients with chronic illness more regularly than did physicians, although none of the 3 types of clinicians routinely provided health education. Possible explanations include training differences, differing roles within a clinic by provider type, or increased clinical demands on physicians. More research is needed to understand the causes of these differences and potential opportunities to increase the delivery of condition-specific education to patients.

## Introduction

Disease self-management is an essential component of care for patients with most chronic conditions. Patients cannot perform daily self-management tasks if they have poor understanding of the disease process, medications used, or the practical tasks they need to accomplish to care for themselves. Health education is, therefore, a vital preventive element in the patient visit. Literature across a variety of specialties indicates that health professionals often fail to provide health education, resulting in ineffective therapies, return visits, and iatrogenic illnesses (1,2). Barriers to the provision of quality health education may include failure of insurance companies to provide reimbursement for education services, lack of time to spend in counseling, and lack of confidence or skill in counseling among health care providers (3–5).

Additionally, there has been little study on whether some types of health care providers are more likely than others to provide health education to patients. Data are scarce on differences between provider types because most large federal clinical databases do not collect data by provider type or on health education. One study used the National Hospital Ambulatory Medical Care Survey (NHAMCS) to compare nurse practitioners with all other types of health professionals and found that nurse practitioners were more likely than physicians to provide preventive health counseling to patients (6). The question of provider differences is especially important as we consider how health care teams should be configured to meet the health care needs of Americans after implementation of the Affordable Care Act in 2014. The objective of our study was to evaluate the rate of health education provision to patients by physicians, physician assistants, and nurse practitioners/certified midwives.

## Methods

We analyzed data from the outpatient department subset of the NHAMCS, which is administered annually by the National Center for Health Statistics at the Centers for Disease Control and Prevention and is designed to collect data on the use and provision of ambulatory care services in hospital emergency and outpatient departments (7). The NHAMCS uses a 4-stage probability sampling procedure to collect nationally representative data. Physicians and hospital staff are trained to complete patient record forms for visits randomly selected via a systematic selection procedure during a 4-week period. The activities of physicians, nurse practitioners/certified midwives (referred to as nurse practitioners in this article), and physician assistants are recorded in this database. Data on demographic characteristics, symptoms, diagnosis of chronic conditions, vital signs, diagnostic and screening services provided, health education provided, treatments implemented, and provider type are collected for each patient on the patient record form. Detailed information about the sampling design and data collection for the NHAMCS is available at http://www.cdc.gov/nchs/ahcd/about_ahcd.htm. Because this study was a secondary analysis of public data from the Centers for Disease Control and Prevention, institutional review board approval was not sought.

We used NHAMCS outpatient department data from 5 years (2005–2009); we selected these 5 years because the items used to collect the data were identical across survey years during that period. Data on 162,012 outpatient department visits during this time were abstracted; we excluded 25,580 visits for the following reasons: the patient made a visit for a new, undiagnosed condition, made a presurgery or a postsurgery visit, or was younger than 18 years, or the visit included care by more than 1 provider type (any combination of physician, physician assistant, and nurse practitioner). 

We included in our analysis visits at which a diagnosis of asthma, chronic obstructive pulmonary disease (COPD), depression, diabetes, hyperlipidemia, hypertension, ischemic heart disease, or obesity was recorded (in NHAMCS question 5a). We also included visits if any of these 8 diagnoses were selected from among the 14 chronic conditions listed in question 5b, “Regardless of the diagnoses written in 5a, does the patient now have . . .” (www.cdc.gov/nchs/data/ahcd/nhamcs100opd_2009.pdf). A list of health education needs for each chronic condition (Box) was developed by using national and international treatment guidelines (8–11), and providers were credited for delivering health education if they documented it in the patient record. Health education was considered to have been provided if it was delivered by the provider or if the provider directed another health professional such as a nurse, social worker, or registered dietician to deliver it during the same visit. Health education provided during a different visit was not included in the analysis because NHAMCS has no mechanism for collecting this information.

**Box. Health Education Needs for Condition Diagnosed**

**Table:** 

Type of Health Education Needed	Diagnosis of Chronic Condition
Asthma education	Asthma
Diet and nutrition	Diabetes, hyperlipidemia, hypertension, ischemic heart disease, obesity
Exercise	Diabetes, obesity
Stress management	Depression
Tobacco use and exposure	Asthma, chronic obstructive pulmonary disease
Weight reduction	Diabetes, obesity

Our final sample consisted of 136,432 records. For each type of health education, we determined the number of patient visits for each type of provider (physician, physician assistant, and nurse practitioner) and computed the percentage of patients who received education by provider type. We used logistic regression models to compute odds ratios and to obtain a statistical test of the null hypothesis that the percentage of patients receiving the indicated type of health education did not vary across provider types. All estimates and *P* values were obtained by using methods that adjust for unequal sampling weights as well as stratification and clustering in the sampling design. We used Stata version 12 (StataCorp LP, College Station, Texas) for all analyses.

## Results

Health education was not routinely provided to patients with a chronic condition. The percentage of visits in which patients received health education did not reach 50% for any combination of health education and provider type (Table). The percentage of visits in which health education was provided ranged from 13.0% (patients with COPD or asthma who were provided counseling on tobacco use and exposure by nurse practitioners) to 42.2% (patients with diabetes or obesity who were provided counseling on exercise by physician assistants).

**Table:** Visits (N = 136,432) to Hospital Outpatient Departments in Which Health Education Was Provided to Patient, by Type of Health Education and Type of Provider^a^

Type of Health Education^b^/Provider Type	Patient Visits, No (%)^c^	Visits That Included Health Education, %	Odds Ratio^d^ (95% CI)	*P* Value
**Asthma education**				
Physician	848 (90.4)	27.8	1 [Reference]	
Physician assistant	22 (3.4)	26.4	0.93 (0.23–3.81)	.92
Nurse practitioner	67 (6.1)	28.6	1.04 (0.40–2.69)	.94
**Diet and nutrition**				
Physician	11,809 (86.9)	27.7	1 [Reference]	
Physician assistant	433 (5.1)	36.5	1.50 (0.90–2.52)	.12
Nurse practitioner	906 (8.1)	38.0	1.60 (1.12–2.31)	.01
**Exercise**				
Physician	6,039 (86.7)	17.6	1 [Reference]	
Physician assistant	194 (4.7)	42.2	3.42 (1.97–5.95)	<.001
Nurse practitioner	521 (8.6)	26.9	1.72 (1.04–2.85)	.03
**Stress management**				
Physician	2,793 (82.8)	20.7	1 [Reference]	
Physician assistant	45 (3.2)	15.9	0.72 (0.23–2.24)	.58
Nurse practitioner	294 (14.1)	41.1	2.68 (1.59–4.51)	<.001
**Tobacco use and exposures**				
Physician	1,482 (88.2)	13.6	1 [Reference]	
Physician assistant	55 (5.0)	36.4	3.62 (2.02–6.48)	<.001
Nurse practitioner	100 (6.7)	13.0	0.95 (0.35–2.54)	.92
**Weight reduction**				
Physician	6,039 (86.7)	13.9	1 [Reference]	
Physician assistant	194 (4.7)	28.8	2.50 (1.40–4.46)	.002
Nurse practitioner	521 (8.6)	24.0	1.96 (1.20–3.19)	.007

Abbreviations: CI, confidence interval.

a Source of data: outpatient department subset of National Hospital Ambulatory Medical Care Survey 2005–2009 (7).

b Asthma education recommended for diagnosis of asthma; diet and nutrition education for diabetes, hyperlipidemia, hypertension, ischemic heart disease, obesity; exercise education for diabetes, obesity; stress management education for depression; tobacco use and exposure education for asthma, chronic obstructive pulmonary disease; weight reduction education for diabetes, obesity.

c Number of visits is unweighted; percentage of visits is weighted and is the percentage of visits for that type of health education. Percentages may not total 100% because of rounding.

d Odds ratios and *P* values calculated by using sampling weights and linearized variance estimation.

Physician assistants or nurse practitioners were more likely to document provision of health education for 5 of the 6 types of health education evaluated (Table). Nurse practitioners were more likely to provide counseling on diet or nutrition (OR = 1.60, *P* = .01) and stress management (OR = 2.68, *P* < .001) than either physicians or physician assistants. Physician assistants were more likely to provide counseling on tobacco use and exposure (OR = 3.62, *P* < .001) for patients with COPD and asthma than were physicians or nurse practitioners. Physician assistants and nurse practitioners were more likely to provide counseling on exercise (physician assistants, OR = 3.42, *P* < .001; nurse practitioners, OR = 1.72, *P* = .03) and weight reduction (physician assistants, OR = 2.50, *P* = .002; nurse practitioners, OR = 1.96, *P* = .007) than were physicians. In the 3 cases in which physician assistants or nurse practitioners were less likely than physicians to deliver health education (counseling on asthma, stress management, and tobacco use and exposure), the differences were not significant.

## Discussion

Physician assistants and nurse practitioners in general provided chronic condition–specific health education to patients with chronic conditions at a higher rate than did physicians. The reasons for this gap are unclear and cannot be explained by using the NHAMCS data set. One potential explanation is that training programs for physician assistants and nurse practitioners may emphasize the provision of health education to patients more than training programs for physicians. Another potential explanation is a division of roles within practices. Because health education is a time-consuming process, physicians may ask patients to schedule a follow-up visit that includes health education with a physician assistant or nurse practitioner instead of delivering the health education himself or herself. Practices may decide that having the physician see new patients and garner the higher new-patient reimbursement while having physician assistants and nurse practitioners see return patients is a favorable allocation of resources (12).

The reasons for the differences among provider type and rates of health education provision may lie with the patients rather than practices or providers. Patients may be less intimidated by physician assistants and nurse practitioners, who do not have the “doctor” title, and they may be more willing to admit their lack of knowledge about their own condition to nonphysician providers. Patients also may request more health education from physician assistants or nurse practitioners because they believe these providers are more likely to avoid medical jargon.

The literature does not provide much more insight into potential differences in the provision of health education among provider types. However, a few studies on diabetes may shed some light. One study of 46 family practices compared practices that had 3 types of staffing combinations: primary care physicians only, family physicians and nurse practitioners, and family physicians and physician assistants (13). Practices staffed with family physicians and nurse practitioners did a better job of managing patients with diabetes than the other 2 types of practices. The study did not clarify how the addition of nurse practitioners caused the improvement in diabetes outcomes.

A second study used Veterans Administration health system data to determine the relationship between staffing characteristics and levels of hemoglobin A1c (HbA1c) among 88,682 patients in 198 outpatient programs (14). The variable of interest was the level of nurse practitioner and physician staffing, and the outcome measure was glycemic control; the study did not include complication, mortality, or morbidity rates. The study found that inclusion of nurse practitioners in the primary care program was significantly associated with lower HbA1c levels by 0.31 percentage points (95% CI, 0.50%– 0.12%) compared with programs that did not include nurse practitioners. Inclusion of physician assistants in the primary care programs was not associated with a significant difference in HbA1c levels. The psychosocial nature of training for nurse practitioners may contribute to the improved treatment of patients with diabetes (15); nurse practitioners may also provide and document health education to patients with diabetes more effectively than physicians (16). Physician assistant training is based on the medical model in structure and philosophy, which may both benefit and limit physician assistant practices. Physician assistants often are drawn to specialty and procedure-oriented medical and surgical specialties such as orthopedics, emergency medicine, and surgical subspecialties and are perhaps better prepared for these specialties than nurse practitioners. However, other factors alone or in combination may account for the observed differences between physician assistants and nurse practitioners, such as type of practice setting or configuration, use of electronic records systems and reminder systems, adherence to clinical practice guidelines, and patient mix, distribution, or scheduling.

Our study did not assess all aspects of diabetes care but did find that nurse practitioners were more likely than physician assistants to provide counseling on diet or nutrition and stress management, whereas physician assistants were more likely to provide health education on smoking cessation, exercise, and weight reduction. Co-management of patients by a physician–nonphysician team in certain patient groups may provide outcomes superior to those provided by physician care alone (17). Furthermore, research is needed on the optimal combination of providers for provision of health education to patients with chronic conditions and the training of providers by provider type.

The ability of patients to participate in disease self-management will become more critical in the future because of the increasing prevalence of chronic disease in the United States. Health education is a key component of patient self-management. Our study has several potential implications. First, curricula in medical schools and residencies should be examined to evaluate whether health education is part of the formal and informal curriculum. Studies have documented the “hidden curriculum” (18,19) in which the formal lessons of patient-centered care and professionalism are undermined by the behavior of mentors who imply that the only important goals of the medical team are to get to the correct diagnosis and complete the daily to-do list. Physician assistant and nurse practitioner training programs may do a better job of teaching the skills of patient-centered care. Or perhaps the longer duration and intensity of physician training programs erode these skills, which are often taught primarily in the first 2 years of training.

Second, our study may argue for increasing the mix of providers in outpatient clinics. If physicians do not have the time, interest, or skill to provide health education, they can include a physician assistant or nurse practitioner on their team, which would allow them to refer patients internally. Having a physician assistant or nurse practitioner on their team may also influence physicians to improve their own skills in health education. Third, our study shows that rates of health education provision in general are low. Even the best educational effort recorded in this study showed that only 42% of patients with diabetes or obesity received education about exercise. Policymakers should consider increasing incentives for providers to deliver chronic condition–specific health education.

This study has several limitations. First, only health education elements documented by providers could be assessed, not what was actually provided during the visit. Thus, our study may underestimate the rate of health education provision. However, during the time of the data collection (2005–2009), health education was part of the evaluation and management coding and billing system. Providers would have had an incentive to document any health education provided to maximize revenue; therefore, we believe that NHAMCS only slightly underestimates the rate at which health education was provided.

Second, although the Centers for Disease Control and Prevention ensures that NHAMCS data collection is representative of primary care delivered in hospital-based clinics throughout the United States, the survey is not representative of primary care as a whole in the United States. Most primary care is delivered in office-based ambulatory care settings, not in hospital-based clinics. One study comparing the provision of health education in NHAMCS and the National Ambulatory Care Medical Survey, which surveys office-based practices, found that physicians who practice in hospital-based clinics were slightly less likely to provide health education (20).

Third, evaluation of the NHAMCS data does not provide details on the health education provided. We were unable to describe the amount or quality of the health education provided, track the health outcomes of the patients who received condition-specific health education, or assess whether the education provided was delivered in a culturally competent manner or provided to patients in their native language.

Significant differences exist between the rates of health education provided by physician assistants and nurse practitioners compared with physicians. The reasons for these differences are unclear but may include differences in training, differences in provider roles within a practice, or in the increased clinical demands on physicians. More research is needed to understand the causes of these differences and potential opportunities to increase the delivery of condition-specific education to patients.
